# Optimizing Malnutrition Risk Detection in Inflammatory Bowel Disease: A Longitudinal Analysis of Serial Nutritional Screening Tools

**DOI:** 10.3390/nu18030383

**Published:** 2026-01-24

**Authors:** Agnese Favale, Valentina Orrù, Nicola Lutzu, Amalia Di Petrillo, Mauro Demurtas, Ivan Ibba, Angelo Italia, Massimo Claudio Fantini, Sara Onali

**Affiliations:** 1 Department of Medical Science and Public Health, University of Cagliari, 09042 Monserrato, Cagliari, Italy; 2 SC Gastroenterologia, Azienda Ospedaliero-Universitaria di Cagliari, 09042 Monserrato, Cagliari, Italy

**Keywords:** nutritional screening tools, malnutrition, GLIM criteria, ESPEN criteria, Fat-Free Body Mass Index, inflammatory bowel disease

## Abstract

**Background:** Malnutrition is frequently under-investigated during remission in patients with Inflammatory Bowel Disease (IBD), despite its significant impact on clinical outcomes and quality of life. This study aimed to evaluate the increase in diagnostic performance of five nutritional screening tools (NSTs) when serially administered to IBD outpatients in sustained clinical remission. **Methods:** In this prospective, single-center cohort study, NSTs were administered, and body composition analysis was performed in IBD patients at baseline and after six months. At both time points, the sensitivity, specificity, predictive values, and accuracy of NSTs in detecting malnutrition and persistent malnutrition per ESPEN and GLIM criteria were evaluated, comparing repeated to single-point assessments. A sensitivity analysis using low FFMI as a reference was also performed. **Results:** Sixty-six IBD patients (32 Crohn’s disease; 34 ulcerative colitis) were enrolled. At baseline, 25.7% and 9% of patients were malnourished according to ESPEN and GLIM criteria, respectively, with 7.5% exhibiting low FFMI. Malnutrition prevalence increased over time to 53%, 16.6%, and 16.6%, respectively. Among NSTs, MUST and SaskIBD-NR consistently exhibited the highest specificity for malnutrition detection at baseline, at 6 months, and for persistent malnutrition for ESPEN, GLIM and low FFMI. Serial (repeated) NST administration markedly improved the specificity of all tools, compared to single-point assessments. **Conclusions:** Serial nutritional screening with MUST or SaskIBD-NR significantly enhances the specificity of malnutrition risk detection in IBD patients in remission, supporting the incorporation of repeated nutritional assessments into clinical practice to offer a practical strategy to enhance screening effectiveness in IBD outpatient care.

## 1. Introduction

Inflammatory bowel diseases (IBD), including ulcerative colitis (UC) and Crohn’s disease (CD), are chronic, relapsing conditions that pose significant nutritional challenges. Malnutrition and low Fat-Free Body Mass Index (FFMI) have been consistently associated with poorer disease outcomes in this population [1,2,3,4,5,6,7]. Patients with IBD face a heightened risk of malnutrition due to a combination of factors, including increased energy demands during disease flares, impaired nutrient absorption, reduced oral intake secondary to gastrointestinal symptoms, dietary restrictions during hospitalizations, and decreased absorptive surface following bowel resections [8,9]. Despite these well-established risks, the prevalence of malnutrition remains alarmingly high in IBD patients, even during disease remission [7], underscoring the critical need for effective nutritional screening [10,11]. The most widely accepted criteria for malnutrition definition, such as those proposed by the ESPEN and GLIM consensus, require objective, time-consuming measures of reduced lean muscle mass [12,13], limiting their routine applicability in an out-patient setting in the absence of a dedicated nutritionist. In light of this, nutritional screening is essential to detect patients who may benefit from further nutritional assessment.

To this end, several nutritional screening tools (NSTs) have been developed. However, their validity is complicated by the lack of a universally accepted gold standard for malnutrition [14], and their performance has not been directly compared in a population of IBD patients in clinical remission, where a disease-related malnutrition might go undetected. Moreover, whether a single time point measurement of the risk may be sufficient to identify patients who will develop malnutrition is unclear. Indeed, there is currently no evidence whether repeated NST administration provides superior identification of nutritional status at follow-up as compared to a single time point evaluation. To address these issues, we conducted a prospective study to evaluate and compare the performance of commonly used NSTs against two malnutrition diagnostic references (namely, the ESPEN and GLIM criteria) in identifying the state of malnutrition and predicting the future or persistent malnutrition risk, and whether serial NST assessments improved diagnostic performance.

Our goal was to identify the most pragmatic and effective approach for nutritional screening in IBD patients, given the aforementioned challenges and based on currently available diagnostic standards.

## 2. Materials and Methods

### 2.1. Patient Selection and Data Collection

This prospective, single-center, longitudinal cohort study included IBD patients followed at the IBD tertiary center of the Policlinico Universitario di Monserrato, Cagliari, Italy. Patients willing to participate and able to sign the informed consent were consecutively enrolled from December 2023 to March 2025. Inclusion criteria required patients to be in sustained clinical remission, defined by a Harvey-Bradshaw Index (HBI) score of <5 for CD and a partial Mayo Score (PMS) of <2 (with rectal bleeding subscore 0) for UC, for at least 6 months as reported in at least two consecutive visits. Exclusion criteria included pregnancy, short bowel syndrome, ileo-pouch anastomosis, presence of an ostomy, current or recent hospitalization, and confounding factors for Bioelectrical Impedance Analysis (BIA) (i.e., pacemaker wearer and ongoing fever).

Demographic and disease-specific characteristics, history of IBD-related surgery, and previous medical history were recorded in a standardized database. Patients were evaluated at baseline (T0) and after six months (T1). Data collected at both time points included C-reactive protein (CRP), fecal calprotectin (FC), vitamin levels, and current medical therapy. HBI and PMS scores were also calculated at T0 and T1.

### 2.2. Malnutrition Risk Assessment

All participants were evaluated at both T0 and T1 by a trained nutritionist together with a gastroenterologist, when anthropometric measurements—including body weight, height, mid-arm and mid-calf circumferences—were recorded, and Body Mass Index (BMI) was calculated. Five NST questionnaires—namely, the Malnutrition Universal Screening Tool (MUST) [15], the Malnutrition Inflammation Risk Tool (MIRT) [16], Mini Nutritional Assessment (MNA) [17], Nutritional Risk Screening 2002 (NRS2002) [18], and the Saskatchewan Inflammatory Bowel Disease—Nutrition Risk Tool (SaskIBD-NR) [19] were administered at both time points (Table S1). Scores from each NST were analyzed dichotomously to classify patients as at risk (+) or not at risk (−) for malnutrition based on specific standard thresholds. Participants were required not to receive any nutritional intervention between T0 and T1. Malnutrition was defined using ESPEN and GLIM criteria, as well as by low FFMI, at both T0 and T1 (Tables S2 and S3).

### 2.3. Body Composition Analysis

Multifrequency (5–250 kHz) BIA was performed at T0 and T1 using the “Human Im Touch” device (DS Medica S.r.l., Milano, Italy). Patients were instructed to remove jewelry and watches, lie in a supine position for 10 min on an empty bladder, and fast for at least 5 h prior to the assessment. They were also advised to avoid physical activity in the previous 12 h and alcohol consumption in the previous 48 h. The data collected included Fat Mass, Fat-Free Mass (FFM), Body Cell Mass (BCM), Body Cell Mass Index (BCMI), and FFMI, derived using Dyetosistem^®^ software (DS Medica S.r.l., version 13.0). A low FFMI was defined as <18 kg/m^2^ in males and <15 kg/m^2^ in females.

### 2.4. Statistical Analysis

Standard descriptive statistics were used to analyze patient characteristics. Categorical variables are presented as counts and percentages. The Saphiro–Wilk test was used to assess the normality of continuous variables. Normally distributed data were expressed as mean ± standard deviation (SD), while skewed data were presented as median and interquartile ranges (IQR). Comparisons between groups were performed using Student’s *t*-test or Chi-squared tests for categorical variables, and the Mann–Whitney U tests for continuous variables, as appropriate. The performance of each NST was evaluated by calculating sensitivity, specificity, positive predictive value (PPV), negative predictive value (NPV), and overall accuracy compared to ESPEN and GLIM as reference. A sensitivity analysis was conducted to compare NSTs’ performance against an objective measure of malnutrition (FFMI). Differences were considered statistically significant at *p* < 0.05. Due to the absence of specific literature on the comparative performance of multiple NSTs against three distinct malnutrition reference standards in IBD patients in sustained clinical remission, a formal a priori sample size calculation was not feasible. Therefore, a target of 50 patients was deemed a pragmatic sample size to conduct an exploratory analysis of NST diagnostic performance within this specific population. All analyses were conducted using Stata BE (version 18.0, StataCorp LLC, College Station, TX, USA).

## 3. Results

### 3.1. Patients and Disease Characteristics

Sixty-six IBD patients were included (32 CD, 34 UC). The median age was 55.5 years (IQR 34–63), with no significant differences between CD and UC. A statistically significant difference was observed in gender distribution between the two groups, with a higher proportion of females in the UC group [11/32 (34%) CD vs. 20/34 (59%) UC; *p* = 0.047]. The median disease duration was 10 years (IQR 6–17). Among CD patients, the most frequent disease location was ileocolonic in 20/32 (63%) patients, followed by isolated ileal in 8/32 (25%), and colonic in 4/32 (13%). Perianal disease was reported in 12/32 (38%) CD patients. Twenty-three out of 34 (67.6%) UC patients had pancolitis, 8/34 (23.5%) proctitis, and 3/34 (8.8%) had left-sided colitis (Table 1).

At baseline, median CRP was 0.8 mg/L (IQR 0–3.8), with no significant differences between the two groups [median CRP CD 0.75 mg/L (IQR 0.15–6.1), UC 1 mg/L (IQR 0–3.1), *p* = 0.559]. Median FC at baseline was 55.2 μg/g (IQR 12.5–327), with no significant differences between CD and UC [median FC CD:76.8 μg/g (IQR 16.5–345.5), UC: 53.05 μg/g (IQR 5–269), *p* = 0.278]. The majority of patients (89.4%) were on advanced therapies, with no significant difference between the groups [CD: 28/32 (88%); UC: 31/34 (91%), *p* = 0.628].

### 3.2. Anthropometric Parameters and Malnutrition Assessment at Baseline

In the entire cohort, median BMI at baseline was 24.05 kg/m^2^ (IQR 21.3–26.4). There was no significant difference in BMI between CD and UC patients. However, a significant difference in the proportion of patients with normal weight (BMI = 18.5–24.9 kg/m^2^) was observed between the groups [21/32 (66%) vs. 14/34 (41%) in CD and UC, respectively; *p* = 0.047], with a higher proportion of CD patients falling into this category. Significant differences were also observed in Body Cell Mass Index (BCMI), with UC patients having lower BCMI values than CD [mean BCMI CD: 10.49 (±2.1); UC: 9.07 (±2.15), *p* = 0.005] (Table 2).

At T1, median BMI was 23.24 kg/m^2^ (IQR 21.56–26.58). A similar proportion of underweight, normal weight, overweight, or obese patients was observed between the groups. Significant differences were observed in %FFM, FFMI and BCMI, with CD patients having higher %FFM values [mean %FFM CD: 80.67 (±9.94); UC: 75.6 (±11.64), *p* = 0.031], higher FFMI [median FFMI CD: 19 (17.25–20.7); UC: 17.38 (15.96–18.74), *p* = 0.025] and higher BCMI [mean BCMI CD: 10.34 (±4.41); UC: 8.65 (±2.63), *p* = 0.03] than UC patients.

At baseline, 17/66 (25.7%) of patients were malnourished according to ESPEN criteria, with a significantly higher prevalence of malnutrition in the UC group [CD: 3/32 (9%); UC:14/34 (41%); *p* = 0.003]. In contrast, six (9%) IBD patients were malnourished according to GLIM [CD: 3/32 (9%); UC:3/34 (9%); *p* = ns], and 5/66 (7.5%) patients had low FFMI [CD: 1/32 (3%); UC:4/34 (11.8%); *p* = ns].

Compared to baseline, a numerically higher proportion of patients were malnourished at T1: 35/66 (53%) and 11/66 (16.6%) according to ESPEN and GLIM, respectively. However, this increase in prevalence was not statistically significant for either ESPEN (*p* = 0.39) or GLIM criteria (*p* = 0.68). Furthermore, no significant difference in malnutrition prevalence was observed at T1 between CD and UC patients [ESPEN CD: 15/32 (47%), UC: 20/34 (59%); *p* = 0.307; GLIM CD: 5/32 (16%), UC: 6/34 (18%); *p* = 0.183]. At T1, 11/66 (16.6%) patients had low FFMI [3/32 (9%) CD, 8/34 (23.5%) UC, *p* = ns] (Table 3). In contrast to ESPEN and GLIM, the proportion of patients with low FFMI significantly increased from 7.5% at T0 to 16.6% at T1 (*p* = 0.002), as reflected by a significant decrease in median FFMI value from T0 to T1 (*p* = 0.002).

### 3.3. Baseline Nutritional Screening Tools Performance to Identify Malnutrition at Baseline According to ESPEN and GLIM

The performance of NSTs administered at baseline in identifying contextual malnutrition (malnutrition at T0) varied considerably depending on the definition of malnutrition adopted. When compared to the ESPEN criteria, MUST and SaskIBD-NR demonstrated the highest specificity (91.84% and 93.88%, respectively) but low sensitivity (29.41% and 5.88%, respectively). When using GLIM criteria as the reference standard, while MUST and SaskIBD-NR consistently reported the highest specificities (93.33% and 98.33%, respectively), all NSTs exhibited higher sensitivities: 83.3% for MUST, MIRT, MNA, and NRS2002, 50% for SaskIBD-NR. Notably, the positive predictive values for these tools were generally low (19.23% to 75.00%) using either ESPEN or GLIM criteria, while negative predictive values were higher, ranging from 95.16% to 98.25% for GLIM across all tools. Overall, accuracy was higher when using GLIM than for ESPEN-defined malnutrition, with MUST and SaskIBD-NR reporting the highest accuracies (75.76% and 71.21% for ESPEN; 92.42% and 93.94% for GLIM, respectively) (Table S4).

### 3.4. Baseline Nutritional Screening Tools Performance to Identify Malnutrition at T1 According to ESPEN and GLIM

Considering that NSTs are intended for risk assessment, we evaluated the capacity of baseline NSTs in identifying individuals at risk for developing malnutrition over a 6-month period independently of nutritional status at baseline. At T1, according to the ESPEN criteria, overall sensitivities were generally low, while MUST and SaskIBD-NR had the highest specificity (93.55% both). When using GLIM criteria as the reference standard, overall sensitivities remained low, while MUST and SaskIBD-NR reported again the highest specificities (87.27% and 94.55%, respectively). Positive predictive values were consistently low, independently of the malnutrition definition adopted. Negative predictive values were low for ESPEN and higher for GLIM, ranging from 80.43% to 85% across all tools. Overall, accuracy was generally low when using either ESPEN or GLIM criteria (Table S5).

### 3.5. Impact of Repeated Nutritional Screening on Diagnostic Performance

**Figure 1:**
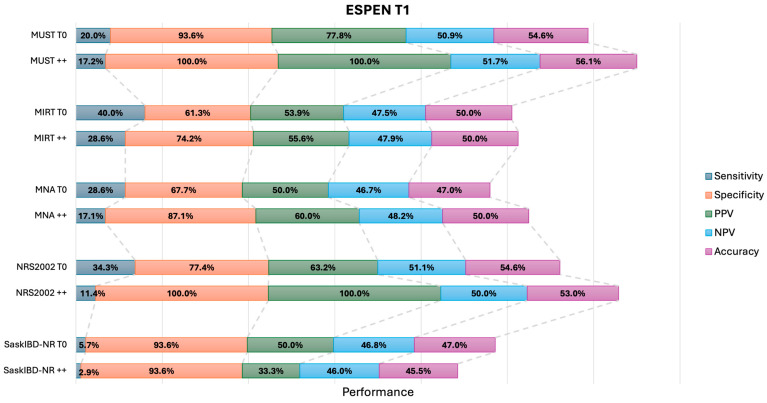
Graphical comparison of performance parameters as indicated (i.e., Sensitivity, Specificity, Positive Predictive Value [PP], Negative Predictive Value [NPV], and Accuracy) of different NSTs resulting in a positive at the single T0 time point or being positive at T0 and T1 (double positive; ++) in detecting ESPEN-defined malnutrition at T1. MUST = Malnutrition Universal Screening Tool; MIRT = Malnutrition Inflammation Risk Tool; MNA = Mini Nutritional Assessment; NRS2002 = Nutritional Risk Screening 2002; SaskIBD-NR = Saskatchewan Inflammatory Bowel Disease—Nutrition Risk Tool.

**Figure 2:**
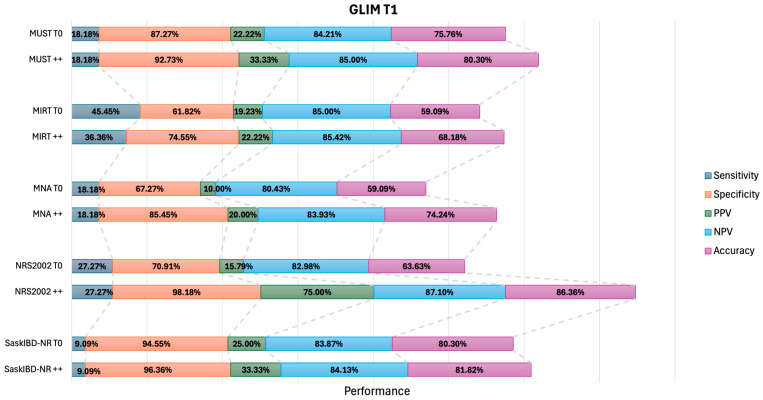
Graphical comparison of performance parameters as indicated (i.e., Sensitivity, Specificity, Positive Predictive Value [PP], Negative Predictive Value [NPV], and Accuracy) of different NSTs resulting in a positive at the single T0 time point or being positive at T0 and T1 (double positive; ++) in detecting GLIM-defined malnutrition at T1. MUST = Malnutrition Universal Screening Tool; MIRT = Malnutrition Inflammation Risk Tool; MNA = Mini Nutritional Assessment; NRS2002 = Nutritional Risk Screening 2002; SaskIBD-NR = Saskatchewan Inflammatory Bowel Disease—Nutrition Risk Tool.

To assess whether repeated administration of the NST improved their capacity in identifying the risk of malnourishment, we compared the performance of two positive NSTs performed at baseline and after six months (NST++), with a single point evaluation at baseline. When adopting the ESPEN criteria, the sensitivity of the NSTs remained generally low. However, notable increases were observed in specificity, with MUST++ and NRS2002++ exhibiting the highest values (100%), followed by SaskIBD-NR ++ at 93.55% (Figure 1). Based on GLIM criteria, the sensitivities also remained low and the specificities high, with MUST++ (92.73%), NRS2002++ (98.18%), and SaskIBD-NR++ (96.36%) exhibiting the highest values (Figure 2). Overall, the accuracies were higher with GLIM compared with the ESPEN criteria. When compared to single administration, the accuracy of all NSTs vs. GLIM, but not vs. ESPEN, increased if administered twice. (Figure 1 and Figure 2 and Table S6).

### 3.6. Diagnostic Performance of Baseline NSTs in Identifying Persistent Malnutrition According to ESPEN and GLIM

To evaluate the ability of baseline NSTs to identify patients who were persistently malnourished, we used a stringent definition of malnutrition, defining patients as positive only if they met ESPEN and GLIM criteria at both baseline and 6-month follow-up. Ten out of 66 (15%) patients were malnourished at both time points according to ESPEN (ESPEN ++), 1/66 (1.5%) patients according to GLIM (GLIM ++). Under this stricter definition, baseline MUST, and SaskIBD-NR exhibited the highest specificity, with MUST demonstrating a specificity of 92.86% against ESPEN++ and SaskIBD-NR reaching 94.64%. When GLIM++ was used as the reference, MUST and SaskIBD-NR maintained strong specificities (87.69% and 95.38%, respectively). Notably, all NSTs achieved NPVs of 100% against GLIM++ Table 4.

### 3.7. Nutritional Screening Tools Performance in Identifying Patients with Low Fat-Free Body Mass Index

To confirm the NST performance against an objective measure of malnutrition, a sensitivity analysis was conducted using low FFMI as the reference standard. At baseline, MUST and SaskIBD-NR were confirmed to be the most specific tools (88.62% and 93.44% specificity, respectively) (Table S7). Accordingly, among NSTs, baseline MUST and SaskIBD-NR showed the highest specificity for predicting the risk of having low FFMI at T1 (89.9% and 94.55%, respectively) (Table S8).

Also, in the context of low FFMI, the performance of repeated NSTs, in terms of specificity, outperformed single administration, at the expense of sensitivity and accuracy (Table S9 and Figure S1).

Finally, MUST and SaskIBD-NR consistently showed the highest specificity also in detecting persistent malnutrition as defined by the presence of low FFMI at both time points (lowFFMI++) (Table S10).

## 4. Discussion

Malnutrition remains a significant and often unrecognized complication in patients with IBD, even during disease remission [9]. Both malnutrition and reduced FFMI have been shown to worsen clinical outcomes, increase morbidity and healthcare costs, and diminish patient quality of life [9,20,21,22,23,24]. Despite these risks, the lack of standardized assessment protocols and limited dedicated personnel in real-world clinical practice frequently leads to malnutrition going undetected [25]. In response to this issue, ESPEN and GLIM consensus recommend systematic nutritional screening, although they do not endorse a specific screening tool due to a lack of clear evidence supporting the superiority of one over another [12,13,26]. The substantial disagreement among NSTs in IBD, as highlighted in a systematic review by Li et al. [27], is not unexpected, given that most of these tools were originally developed for different patient populations and settings, such as hospitalized individuals or those with non-IBD related conditions [15,16,17,18]. In response to the need for a screening instrument designed for the IBD population, the SaskIBD-NR was created to incorporate relevant risk factors for malnutrition [19]. However, the process of validating NSTs against different malnutrition criteria—such as ESPEN, GLIM, or other locally adopted standards—represents a major limitation when comparing their effectiveness [28]. As a result, a screening tool that shows good performance with one malnutrition definition may perform differently, or even inadequately, when tested against another. This variability fundamentally undermines both the broad validity and the generalizability of these tools. Accordingly, major guidelines now advise clinicians to select screening tools based on screening goals, clinical context, and available resources, rather than relying on a single universal tool [29].

Historically, nutritional assessment has focused more on CD than UC, possibly due to the pan-enteric involvement of Crohn’s disease and the increasing evidence that specific diets may ameliorate intestinal inflammation and reduce post-surgical complications in CD, while comparable findings in UC remain scarce. [30,31,32,33,34,35,36,37,38,39,40,41] However, in our study, malnutrition prevalence was similarly high in both diseases. This finding suggests that UC patients, often less prioritized for nutritional screening, face comparable risks and warrants systematic nutritional assessment to prevent overlooked malnutrition.

In our prospective cohort, malnutrition prevalence increased over time. This might indicate reclassification effects due to repeated nutritional screening, rather than a clear and definitive decline in nutritional status, though one does not exclude the possibility of the other. In particular, malnutrition prevalence ranged from 9% (GLIM) to 25% (ESPEN) at baseline, rising to 16% and 53%, respectively, after six months.

These findings are consistent with the study by Bezzio et al. [42], in which the prevalence of GLIM malnourished patients at baseline was 13.3%. Discrepancies with other studies—like the higher ESPEN-defined rates seen by Gold et al. [43], (36% vs. our 25.7%)—can likely be attributed to differences in patient populations, as patients with active IBD were included in their cohort, evidenced by the fact that over 30% reported diarrhea. An Italian study by Fiorindi et al. [44], involving 62 complicated IBD patients requiring surgery, reported a higher GLIM-defined malnutrition prevalence (40%) than observed in our outpatient cohort, a difference likely attributable to the pre-surgical context and the inclusion of patients with ostomies [44]. Finally, a recent study [45] comparing IBD patients to healthy controls, found higher malnutrition detection rates using GLIM criteria compared to ESPEN—a finding likely attributable to the incorporation of active disease measures in the GLIM algorithm [4]. Accordingly, we observed a significant increase in patients with low FFMI between T0 and T1, while the numerical increase in ESPEN and GLIM malnourished patients did not reach statistical significance. This discrepancy is particularly noteworthy as it suggests that ESPEN and GLIM criteria may not adequately reflect the progressive, detrimental changes in body composition over time.

When evaluating tool performance, we observed that all NSTs were more accurate in identifying GLIM-defined malnutrition compared to the ESPEN criteria. Specifically, MUST and SaskIBD-NR repeatedly showed the highest specificity, not only in cross-sectional analysis but also as predictors of future malnutrition and low FFMI. The high specificity of MUST and SaskIBD-NR confirms the findings of recent studies in different IBD populations and clinical settings, including pre-operative and active disease [22,42,44]. However, none of these studies specifically computed their validity against ESPEN and low FFMI. It is also notable that, in our sensitivity analysis using low FFMI as a reference for malnutrition, the general trends of tool performance persisted: MUST and SaskIBD-NR were again the most specific and reliable screening tools for identifying patients at risk of reduced muscle mass. This consistency across malnutrition standard definitions and an objective benchmark like FFMI strengthens the applicability of these tools for routine outpatient care and underscores their pragmatic value when access to direct body composition assessment is limited.

Our data are further supported by a systematic review of the literature that found a significant association between MUST and FFMI [27], although Csontos et al. [46] reported that MUST failed to accurately identify patients with low FFMI at risk for sarcopenia. However, their study population differed significantly from ours in terms of disease activity and UC disease extent, having lower rates of pancolitis.

When exploring the ability of NSTs to predict persistent malnutrition, MUST and SaskIBD-NR were confirmed to be the best in terms of specificity and accuracy. This was true for ESPEN, GLIM, and low FFMI. Of note, all NSTs showed a sensitivity of 100% for patients persistently malnourished according to GLIM, but not ESPEN.

Importantly, our longitudinal design indicates that nutritional status in IBD is dynamic. Indeed, in the absence of targeted intervention, malnutrition may substantially worsen over time even during remission periods, underscoring the critical need for systematic surveillance rather than a “one-off” approach. Our study supports this strategy by demonstrating that serial (repeated) administration of NSTs—unlike single-point assessment—markedly increases specificity and overall diagnostic confidence, irrespective of the malnutrition reference standard, though the accuracy using ESPEN as a reference definition remained low. To our knowledge, this is the first prospective study in IBD patients to show the incremental value of repeated nutritional screening assessments in improving specificity and accuracy for identifying patients not at risk of malnutrition. This enhanced performance is particularly valuable for reliably identifying patients who are not at risk of developing malnutrition, thereby optimizing resource allocation by reducing unnecessary further assessments. Indeed, by demonstrating that serial use of validated NSTs, especially MUST and SaskIBD-NR enhances specificity—without sacrificing feasibility—our study introduces a pragmatic workflow that could be readily incorporated in outpatient settings, allowing for a more reliable identification of patients who are unlikely to become malnourished over time and at the same time identifying those who deserve a deeper evaluation of the nutritional status. This approach is particularly advantageous in contexts where resources are limited, yet the burden of malnutrition remains substantial, leading to the optimization of referral pathways and reducing unnecessary interventions.

Although the single-center design and relatively small sample size might limit the definitive applicability of our results to broader clinical practice, our findings provide strong hypothesis-generating evidence. While we cannot yet conclusively claim an immediate change in routine care, these results have the potential for improving the efficiency and effectiveness of malnutrition screening in IBD. This warrants further investigation to conclusively validate these findings and establish their widespread clinical utility. Notably, our results reveal that while specificity is significantly enhanced by repeated screening, sensitivity remains modest for all tools—especially with ESPEN as the definition reference. This highlights the constant need for clinical judgment, and in high-risk scenarios or where the clinical consequences of missed malnutrition might be serious, such as in a pre-surgical setting, a lower threshold for comprehensive evaluation or use of objective body composition techniques (e.g., FFMI) should be maintained.

A major strength of this study is its prospective design, the longitudinal head-to-head assessment of nutritional risk with multiple validated NSTs, and the incorporation of both established (ESPEN, GLIM) and objective (FFMI) malnutrition criteria. This methodology allowed comprehensive and clinically relevant comparisons of different NSTs missing in the previously published literature. Furthermore, our demonstration of serial screening’s added value provides new evidence that may inform everyday clinical practice.

However, some limitations should be acknowledged. The single-center design and focus on outpatients in clinical remission may limit generalizability to other populations, such as those with active disease, hospitalized patients, or individuals undergoing surgical interventions. However, in these settings, where malnutrition has a significant implication on disease outcomes, nutritional assessment may be preferred over screening. Nevertheless, our specific focus on patients in remission aligns with the most recent British guidelines, which advocate for the review of nutritional status as part of monitoring remission in CD, highlighting the persistent importance of nutritional surveillance even in stable disease [47]. Furthermore, in the present study, FFMI was derived using BIA measurements rather than Dual Energy X-ray Absorptiometry (DEXA), which is considered the gold standard for body composition analysis. However, this methodological choice was central to our study’s aim of screening in routine outpatient settings, where highly expensive, radiation-exposing, or not readily available methods like DEXA are impractical. The use of BIA for FFMI derivation ensures that our findings remain feasible for daily clinical practice.

Finally, nutritional interventions between screening points were controlled in this study, whereas real-world practice may involve varied management that could influence outcomes.

## 5. Conclusions

In summary, this longitudinal cohort study provides new insights into the comparative effectiveness of widely used NSTs in assessing malnutrition risk among IBD patients in clinical remission. Our findings indicate that, while malnutrition and low FMMI remain prevalent, they might even increase over time despite clinical disease control. In this setting, we found that serial administration of NSTs, particularly MUST and SaskIBD-NR, significantly enhances specificity in detecting and tracking malnutrition risk in IBD outpatients in remission. This novel finding supports the integration of repeated, rather than single-point, nutritional risk assessments into routine clinical care. Implementing serial screening could optimize resource allocation, ensure earlier identification of at-risk patients, and ultimately contribute to improved health outcomes in IBD management. Future research should validate these findings in broader and more diverse IBD populations, as well as evaluate the cost-effectiveness and clinical impact of implementing serial NST use in everyday practice.

## Figures and Tables

**Table 1:** Demographics and disease characteristics.

	IBD (*n* = 66)	CD (*n* = 32)	UC (*n* = 34)	*p*
Age, median (IQR)	55.5 (34–63)	46.5 (33–62.5)	57 (39–63)	0.281
Gender (F), *n* (%)	31 (47)	11 (34)	20 (59)	**0.047**
Active smoking, *n* (%)	13 (19.7)	11 (34)	2 (6)	
Disease duration, yrs, median (IQR)	10 (6–17)	7 (4–16)	10 (6–17)	0.115
CD location *n* (%)				
L1		8 (25)		
L2		4 (13)		
L3		20 (63)		
CD behavior *n* (%)				
B1		18 (56)		
B2		10 (31)		
B3		4 (13)		
Perianal disease, *n* (%)		12 (38)		
Previous surgery, *n* (%)		20 (63)		
UC extension, *n* (%)				
E1			8 (23.5)	
E2			3 (8.8)	
E3			23 (67.6)	
CRP, median (IQR)	0.8 (0–3.8)	0.75 (0.15–6.1)	1 (0–3.1)	
FC, median (IQR)	55.2 (12.5–327)	76.8 (16.5–345.5)	53.05 (5–269)	
Conventional therapy				
Mesalamine, *n* (%)	35 (53)	8 (25)	27 (79)	
Immunosuppressants, *n* (%)	3 (4.5)	2 (6)	1 (3)	
Steroids, *n* (%)	6 (9)	2 (6)	4 (12)	
Advanced Therapies, *n* (%)	59 (89.4)	28 (88)	31 (91)	0.628
Infliximab	32 (48.5)	13 (41)	19 (56)	
Adalimumab	8 (12)	7 (22)	1 (3)9	
Vedolizumab	9 (13.6)	3 (9)	6 (18)	
Ustekinumab	6 (9)	4 (13)	2 (6)	
Upadacitinib	2 (3)	0	2 (6)	
Risankizumab	1 (1.5)	1 (3)	n.a.	
Tofacitinib	1 (1.5)	n.a.	1 (3)	

CD = Crohn’s disease, UC = Ulcerative Colitis, IBD = Inflammatory Bowel Disease, L1 = ileal, L2 = ileo-colonic, L3 = colonic, B1 = non-stricturing, non-penetrating, B2 = stricturing, B3 = penetrating, E1 = proctitis, E2 = left-sided colitis, E3 = pancolitis, n.a.= not applicable. Bold font indicates statistical significance (*p* < 0.05).

**Table 2:** Anthropometric characteristics and malnutrition prevalence at baseline (T0).

	IBD (*n* = 66)	CD (*n* = 32)	UC (*n* = 34)	*p*-Value
BMI, median (IQR)	24.05 (21.3–26.4)	24.05 (21.55–26.05)	23.55 (20.8–26.5)	0.802
Underweight, *n* (%) (BMI < 18.5 km/m^2^)	5 (7.6)	0	5 (15)	
Normal weight, *n* (%) (BMI 18.5–24.9 km/m^2^)	35 (53)	21 (66)	14 (41)	**0.047**
Overweight, *n* (%) (BMI 25–29.9 km/m^2^)	21 (31.8)	10 (31)	11 (32)	0.923
Obese, *n* (%) (BMI > 29.9 km/m^2^)	5 (7.6)	1 (3)	4 (12)	
Weight, kg, median (IQR)	63.5 (57.8–76.5)	66 (58.05–77.85)	61.75 (55–75.3)	0.423
%Fat, mean (±SD)	19.68 (±11.7)	18.12 (±10.97)	21.14 (±12.3)	0.851
%FFM, mean (±SD)	80.17 (±11.65)	81.8 (±10.88)	78.6 (±12.3)	0.138
FFMI, median (IQR)	18.6 (16.9–20.8)	19.35 (17.9–21.3)	17.75 (16.7–19.9)	**0.038**
FFMI < 17 (M), *n* (%)	4 (6)	1 (3)	3 (8.8)	
FFMI < 15 (F), *n* (%)	1 (1.5)	0	1 (3)	
BCMI, mean (±SD)	9.75 (±2.27)	10.49 (±2.18)	9.07 (±2.15)	**0.005**
ESPEN malnourished, *n* (%)	17 (25.7)	3 (9)	14 (41)	**0.003**
GLIM malnourished, *n* (%)	6 (9)	3 (9)	3 (9)	

CD = Crohn’s disease, UC = Ulcerative Colitis, IBD = Inflammatory Bowel Disease, BMI = Body Mass Index, FFM = Fat-Free Mass, FFMI = Fat-Free Mass Index, BCMI = Body Cell Mass Index, Bold font indicates statistical significance (*p* < 0.05).

**Table 3:** Anthropometric characteristics and malnutrition prevalence at T1.

	IBD (*n* = 66)	CD (*n* = 32)	UC (*n* = 34)	*p*-Value
BMI, median (IQR)	23.24 (21.56–26.58)	23 (21.59–26.41)	23.7 (21.46–26.64)	0.938
Underweight, *n* (%) (BMI < 18.5 km/m^2^)	2 (3)	0	2 (6)	
Normal weight, *n* (%) (BMI 18.5–24.9 km/m^2^)	39 (59)	22 (68.8)	17 (50)	0.122
Overweight, *n* (%) (BMI 25–29.9 km/m^2^)	21 (31.8)	9 (28)	12 (35)	0.532
Obese, *n* (%) (BMI > 29.9 km/m^2^)	4 (6)	1 (3)	3 (9)	
Weight, kg, median (IQR)	64.7 (58–77)	65.25 (59.5–76.15)	61.45 (54–77)	0.488
%Fat, mean (±SD)	21.9 (±11.07)	19.33 (±9.94)	24.4 (±11.65)	0.968
%FFM, mean (±SD)	78 (11.07)	80.67 (9.94)	75.6 (11.64)	**0.031**
FFMI, median (IQR)	18 (16.5–19.9)	19 (17.25–20.7)	17.38 (15.96–18.74)	**0.025**
FFMI < 17 (M), *n* (%)	7 (10.6)	3 (9)	4 (12)	
FFMI < 15 (F), *n* (%)	4 (6)	0	4 (12)	
BCMI, mean (±SD)	9.47 (±3.68)	10.34 (±4.41)	8.65 (±2.63)	**0.03**
ESPEN malnourished, *n* (%)	35 (53)	15 (47)	20 (59)	0.307
GLIM malnourished, *n* (%)	11 (16.6)	5 (16)	6 (18)	0.183

CD = Crohn’s disease, UC = Ulcerative Colitis, IBD = Inflammatory Bowel Disease, BMI = Body Mass Index, FFM = Fat-Free Mass, FFMI = Fat-Free Mass Index, BCMI = Body Cell Mass Index, Bold font indicates statistical significance (*p* < 0.05).

**Table 4:** Diagnostic performance of baseline (T0) NSTs in identifying persistent malnutrition according to ESPEN and GLIM.

ESPEN ++					
	Sensitivity	Specificity	PPV	NPV	Accuracy
**MUST T0**	50.00%	92.86%	55.56%	91.23%	86.36%
**MIRT T0**	60.00%	64.29%	23.08%	90.00%	63.64%
**MNA T0**	60.00%	75.00%	30.00%	91.30%	72.73%
**NRS2002 T0**	70.00%	78.57%	36.84%	93.62%	77.27%
**SaskIBD-NR T0**	10.00%	94.64%	25.00%	85.48%	81.82%
**GLIM ++**					
	**Sensitivity**	**Specificity**	**PPV**	**NPV**	**Accuracy**
**MUST T0**	100.00%	87.69%	11.11%	100.00%	87.88%
**MIRT T0**	100.00%	61.54%	3.85%	100.00%	62.12%
**MNA T0**	100.00%	70.77%	5.00%	100.00%	71.21%
**NRS2002 T0**	100.00%	72.31%	5.26%	100.00%	72.23%
**SaskIBD-NR T0**	100.00%	95.38%	25.00%	100.00%	95.45%

MUST = Malnutrition Universal Screening Tool, MIRT = Malnutrition Inflammation Risk Tool, MNA = Mini Nutritional Assessment, NRS2002 = Nutritional Risk Screening 2002, SaskIBD-NR = Saskatchewan Inflammatory Bowel Disease—Nutrition Risk Tool. ESPEN ++ = patients malnourished according to ESPEN at both time points; GLIM ++ = patients malnourished according to GLIM at both time points

## Data Availability

The original contributions presented in this study are included in the article and Supplementary Materials. Further inquiries can be directed to the corresponding author as indicated in the submitting system.

## Supplementary Materials

The following supporting information can be downloaded at: https://www.mdpi.com/article/10.3390/nu18030383/s1, Table S1: Nutritional Screening Tools; Table S2: ESPEN criteria; Table S3: GLIM criteria; Table S4: Baseline (T0) Nutritional Screening Tools performance in identify malnutrition at T0 according to ESPEN and GLIM; Table S5: Baseline (T0) Nutritional Screening Tools performance in identify malnutrition at T1 according to ESPEN and GLIM; Table S6: Diagnostic performance of repeated NSTs in identify malnutrition at T1 according to ESPEN and GLIM; Table S7: Baseline (T0) Nutritional Screening Tools performance in identify malnutrition at T0 according to low FFMI; Table S8: Baseline (T0) Nutritional Screening Tools performance in identify malnutrition at T1 according to low FFMI; Table S9: Diagnostic performance of repeated NSTs in identify malnutrition at T1 according to low FFMI; Table S10: Diagnostic Performance of Baseline (T0) NSTs in Identifying Persistent Malnutrition according to low FFMI; Figure S1: Change in performance of single vs repeated (++) NSTs in detecting low FFMI at T1.
