# Supplementary material for: Optimizing Malnutrition Risk Detection in Inflammatory Bowel Disease: A Longitudinal Analysis of Serial Nutritional Screening Tools
Source: Nutrients. 2026 Jan 24;18(3):383. doi: 10.3390/nu18030383 (PMC12899848; doi:10.3390/nu18030383)

Supplementary Table S1. Nutritional Screening Tools

	BMI	Weight loss	Reduced Food Intake	Severity of Disease	CRP	GI symptoms	Age
**MUST**	18.5-20	5-10% in 3-6 months	Yes in the last 5 days or pts acutely ill				
	<18.5	>10% in 3-6 months					
**MIRT**	18.5-20	5-10% in 3 months			5-50 mg/L		
	<18.5	≥10% in 3 months			≥50 mg/L		
**MNA**	≥23	No weight loss	No decrease	No psychological problem<br><br>Normal mobility			
	21-22	1-3 kg last 3 months	Moderate decrease	Mild dementia<br><br>Able to get out of bed – not goes out			
	19-20	Not known	Severe decrease	Severe dementia or depression<br><br>Bed or chair bound			
	<19	>3 kg last 3 months					
**NRS-2002**	x	>5% in 2 months	50-75% of normal in previous week	Hip fracture, COPD, cirrhosis, diabetes, oncology, hemodialysis			>70yrs
	18.5-20	>5% in 2 months	25-60% of normal in previous week	Major abdominal surgery, stroke, hematologic malignancy			
	<18.5	>5% in 1 month or >15% in 3 months	0-25% of normal in previous week	Head injury, Bone marrow transplantation, ICU patients			
**SaskIBD_NR**		2.3-4.5 kg in 1 month	Eating less for poor appetite			1-2	
		4.5-7 kg in 1 month	Food restriction			>3	
		>7 kg in 1 month					

MUST=Malnutrition Universal Screening Tool, MIRT=Malnutrition Inflammation Risk Tool, MNA=Mini Nutritional Assessment, NRS-2002=Nutritional Risk Screening 2002, SaskIBD-NR=Saskatchewan Inflammatory Bowel Disease – Nutrition Risk Tool

Supplementary Table S2. ESPEN criteria

	BMI	Weight loss %	Low BMI	FFMI
**ESPEN**	< 18,5	10% indefinite time	<20 kg/m^2 if <70 years of age	<15 Kg/m^2 in woman
		>5% over the last 3 months	<22 kg/m^2 if older	<17 Kg/m^2 in men

BMI <18,5 Kg/m2 or Weight loss % plus, either low BMI or FFMI are required for diagnosis; BMI=Body Mass Index, FFMI=Fat-Free Body Mass Index

Supplementary Table S3. GLIM criteria

	**Phenotypic criteria**			**Etiologic criteria**	
	**Low BMI**	**Weight loss %**	**Reduced muscle mass**	**Reduced food intake or assimilation**	**Inflammation**
**GLIM**	<20 if < 70 years	>5% within past 6 months	Assessed through validated body composition measuring techniques	≤ 50% of ER for > 1 week,	Acute disease/injury or chronic disease-related
	<22 if >70 years	>10% beyond 6 months		Any reduction for >2 weeks	
				Any chronic GI condition that adversely impacts food assimilation or absorption	

GI=gastro-intestinal; ER=energy requirements

Supplementary Table S4. Baseline (T0) Nutritional Screening Tools performance in identify malnutrition at T0 according to ESPEN and GLIM

ESPEN T0					
	**Sensitivity**	**Specificity**	**PPV**	**NPV**	**Accuracy**
**MUST T0**	29.41%	91.84%	55.56%	78.95%	75.76%
**MIRT T0**	52.94%	65.31%	34.62%	80.0%	62.12%
**MNA T0**	47.06%	75.51%	40.00%	80.43%	68.18%
**NRS2002 T0**	41.18%	75.51%	36.84%	78.72%	66.67%
**SaskIBD-NR T0**	5.88%	93.88%	25.00%	74.19%	71.21%
**GLIM T0**					
	**Sensitivity**	**Specificity**	**PPV**	**NPV**	**Accuracy**
**MUST T0**	83.33%	93.33%	55.56%	98.25%	92.42%
**MIRT T0**	83.33%	65.00%	19.23%	97.50%	66.67%
**MNA T0**	83.33%	75.00%	25.00%	97.83%	75.76%
**NRS2002 T0**	83.33%	76.67%	26.32%	97.87%	72.27%
**SaskIBD-NR T0**	50.00%	98.33%	75.00%	95.16%	93.94%

MUST=Malnutrition Universal Screening Tool, MIRT=Malnutrition Inflammation Risk Tool, MNA=Mini Nutritional Assessment, NRS-2002=Nutritional Risk Screening 2002, SaskIBD-NR=Saskatchewan Inflammatory Bowel Disease – Nutrition Risk Tool

Supplementary Table S5. Baseline (T0) Nutritional Screening Tools performance in identify malnutrition at T1 according to ESPEN and GLIM

ESPEN T1					
	**Sensitivity**	**Specificity**	**PPV**	**NPV**	**Accuracy**
**MUST T0**	20.00%	93.55%	77.78%	50.88%	54.55%
**MIRT T0**	40.00%	61.29%	53.85%	47.50%	50.00%
**MNA T0**	28.57%	67.74%	50.00%	46.65%	46.97%
**NRS2002 T0**	34.29%	77.42%	63.16%	51.06%	54.55%
**SaskIBD-NR T0**	5.71%	93.55%	50.00%	46.77%	46.97%
**GLIM T1**					
	**Sensitivity**	**Specificity**	**PPV**	**NPV**	**Accuracy**
**MUST T0**	18.18%	87.27%	22.22%	84.21%	75.76%
**MIRT T0**	45.45%	61.82%	19.23%	85.00%	59.09%
**MNA T0**	18.18%	67.27%	10.00%	80.43%	59.09%
**NRS2002 T0**	27.27%	70.91%	15.79%	82.98%	63.63%
**SaskIBD-NR T0**	9.09%	94.55%	25.00%	83.87%	80.30%

MUST=Malnutrition Universal Screening Tool, MIRT=Malnutrition Inflammation Risk Tool, MNA=Mini Nutritional Assessment, NRS-2002=Nutritional Risk Screening 2002, SaskIBD-NR=Saskatchewan Inflammatory Bowel Disease – Nutrition Risk Tool

Supplementary Table S6. Diagnostic performance of repeated NSTs in identify malnutrition at T1 according to ESPEN and GLIM

**ESPEN T1**					
	**Sensitivity**	**Specificity**	**PPV**	**NPV**	**Accuracy**
**MUST ++**	17.24%	100.00%	100.00%	51.67%	56.06%
**MIRT ++**	28.57%	74.19%	55.56%	47.92%	50.00%
**MNA ++**	17.14%	87.10%	60.00%	48.21%	50.00%
**NRS2002 ++**	11.43%	100.00%	100.00%	50.00%	53.03%
**SaskIBD-NR ++**	2.86%	93.55%	33.33%	46.03%	45.45%
**GLIM T1**					
	**Sensitivity**	**Specificity**	**PPV**	**NPV**	**Accuracy**
**MUST ++**	18.18%	92.73%	33.33%	85.00%	80.30%
**MIRT ++**	36.36%	74.55%	22.22%	85.42%	68.18%
**MNA ++**	18.18%	85.45%	20.00%	83.93%	74.24%
**NRS2002 ++**	27.27%	98.18%	75.00%	87.10%	86.36%
**SaskIBD-NR ++**	9.09%	96.36%	33.33%	84.13%	81.82%

MUST=Malnutrition Universal Screening Tool, MIRT=Malnutrition Inflammation Risk Tool, MNA=Mini Nutritional Assessment, NRS-2002=Nutritional Risk Screening 2002, SaskIBD-NR=Saskatchewan Inflammatory Bowel Disease – Nutrition Risk Tool

Supplementary Table S7. Baseline (T0) Nutritional Screening Tools performance in identify malnutrition at T0 according to low FFMI

LOW FFMI T0					
	**Sensitivity**	**Specificity**	**PPV**	**NPV**	**Accuracy**
**MUST T0**	40%	88.52%	22.22%	94.74%	84.85%
**MIRT T0**	60%	62.30%	11.54%	95%	62.12%
**MNA T0**	40%	70.49%	10%	93.48%	68.18%
**NRS2002 T0**	40%	72.13%	10.53%	93.62%	69.70%
**SaskIBD-NR T0**	0%	93.44%	0%	91.94%	86.36%

MUST=Malnutrition Universal Screening Tool, MIRT=Malnutrition Inflammation Risk Tool, MNA=Mini Nutritional Assessment, NRS-2002=Nutritional Risk Screening 2002, SaskIBD-NR=Saskatchewan Inflammatory Bowel Disease – Nutrition Risk Tool

Supplementary Table S8. Baseline (T0) Nutritional Screening Tools performance in identify malnutrition at T1 according to low FFMI

LOW FFMI T1					
	**Sensitivity**	**Specificity**	**PPV**	**NPV**	**Accuracy**
**MUST T0**	27.27%	89.9%	33.33%	85.96%	78.79%
**MIRT T0**	45.45%	61.82%	19.23%	85%	59.09%
**MNA T0**	45.45%	72.73%	25%	86.96%	68.18%
**NRS2002 T0**	45.45%	74.55%	26.32%	87.23%	69.70%
**SaskIBD-NR T0**	9%	94.55%	25%	83.87%	80.30%

MUST=Malnutrition Universal Screening Tool, MIRT=Malnutrition Inflammation Risk Tool, MNA=Mini Nutritional Assessment, NRS-2002=Nutritional Risk Screening 2002, SaskIBD-NR=Saskatchewan Inflammatory Bowel Disease – Nutrition Risk Tool

Supplementary Table S9. Diagnostic performance of repeated NSTs in identify malnutrition at T1 according to low FFMI

LOW FFMI T1					
	**Sensitivity**	**Specificity**	**PPV**	**NPV**	**Accuracy**
**MUST ++**	18.18%	92.73%	33.33%	85%	80.30%
**MIRT ++**	27.27%	72.73%	16.67%	83.33%	65.15%
**MNA ++**	18.18%	85.45%	20%	83.93%	74.24%
**NRS2002 ++**	9%	94.55%	25%	83.87%	80.22%
**SaskIBD-NR ++**	0%	94.55%	0%	82.54%	78.78%

MUST=Malnutrition Universal Screening Tool, MIRT=Malnutrition Inflammation Risk Tool, MNA=Mini Nutritional Assessment, NRS-2002=Nutritional Risk Screening 2002, SaskIBD-NR=Saskatchewan Inflammatory Bowel Disease – Nutrition Risk Tool

Supplementary Table S10. Diagnostic Performance of Baseline (T0) NSTs in Identifying Persistent Malnutrition according to low FFMI

**LOW FFMI ++**					
	**Sensitivity**	**Specificity**	**PPV**	**NPV**	**Accuracy**
**MUST T0**	50.00%	88.71%	22.22%	96.49%	86.36%
**MIRT T0**	75.00%	62.90%	11.54%	97.50%	63.64%
**MNA T0**	50.00%	70.97%	10.00%	95.65%	69.70%
**NRS2002 T0**	50.00%	72.58%	10.53%	95.74%	71.21%
**SaskIBD-NR T0**	0.00%	93.55%	0.00%	93.55%	87.88%

MUST=Malnutrition Universal Screening Tool, MIRT=Malnutrition Inflammation Risk Tool, MNA=Mini Nutritional Assessment, NRS-2002=Nutritional Risk Screening 2002, SaskIBD-NR=Saskatchewan Inflammatory Bowel Disease – Nutrition Risk Tool

Supplementary Figure S1. Change in performance of single vs repeated (++) NSTs in detecting low FFMI at T1. MUST=Malnutrition Universal Screening Tool; MIRT=Malnutrition Inflammation Risk Tool; MNA=Mini Nutritional Assessment; NRS-2002=Nutritional Risk Screening 2002; SaskIBD-NR=Saskatchewan Inflammatory Bowel Disease – Nutrition Risk Too

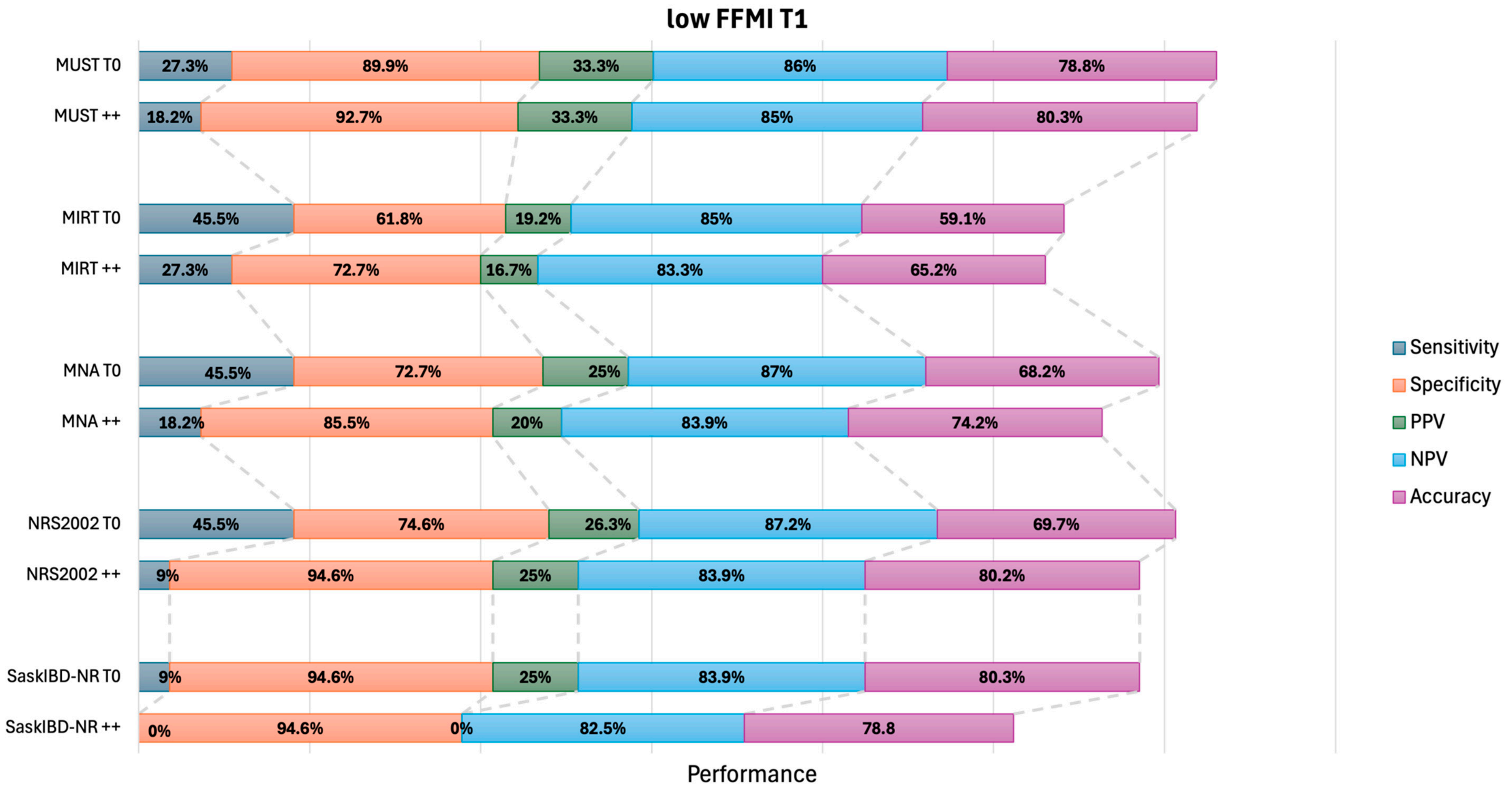

Supplement: Supplementary file 1 [file nutrients-18-00383-s001.zip › nutrients-4100442-supplementary.pdf]
